# Prevalence of Epilepsy in Iran: A Meta-Analysis and Systematic Review

**Published:** 2014

**Authors:** Kourosh SAYEHMIRI, Hamed TAVAN, Fatemeh SAYEHMIRI, Iman MOHAMMADI, Kristin V. CARSON

**Affiliations:** 1 Psychosocial Injuries Research Center, Ilam University of Medical Sciences, Ilam, Iran; 2 Departments of Community Medicine, Faculty of Medicine, Ilam University of Medical Sciences, Ilam, Iran; 3 Student Research Committee, Ilam University of Medical Sciences, Ilam, Iran; 4 The Clinical Practice Unit, The Queen Elizabeth Hospital, Adelaide, Australia

**Keywords:** Epilepsy, Iran, Prevalence, Meta-analysis, Systematic review

## Abstract

**Objective:**

Epilepsy is one of the most common diseases in Iran contributing to an array of health problems. In light of this, the aim of the present study is to examine the prevalence of epilepsy in Iran through a systematic review and meta-analysis.

**Materials & Methods:**

A systematic search of several databases including PubMed, scientific information databases, Google, Google scholar, Elsevier and Scopus was conducted in June 2013. Observational studies were considered for inclusion if they were published in Iranian and examined epilepsy prevalence and/or related risk factors. Meta-analysis was conducted using a random effect model with the DerSimonian/Laird method. Heterogeneity was examined using the Breslow- Day test and inconsistency using the I2 statistic.

**Results:**

A total of 45 studies were identified from the search strategy. Of these, nine published manuscripts with a total of 7,723 participants were included within the review. The pooled prevalence of epilepsy in Iran was estimated to be around 5% (95% confident interval (CI) 2 to 8). For each region the prevalence of epilepsy in central, northern and eastern Iran were 5% (95%CI 2 to 8), 1% (95%CI -1 to 3) and 4% (95%CI 3 to 11) respectively. The most common risk factors in order of prevalence were somatic diseases 39% (95%CI 15 to 62), convulsion 38% (95%CI 11 to 65), mental diseases 36% (95%CI 15 to 95) and hereditary development 26% (95%CI 9 to 42). A meta-regression model identified a declining trend in the prevalence of epilepsy within Iran for the last decade.

**Conclusion:**

Pooled analyses from the nine included publications in this review estimate the prevalence of epilepsy in Iran to be around 5%. Although this result is much higher than rates in other countries, a declining trend in prevalence over the past decade was also identified.

## Introduction

Epilepsy is a one of the most common neurological disorders of childhood leading to excessive and abnormal neuronal activity in the brain (1). Status epilepticus has been defined as a tonic-clonic seizure lasting more than 30 minutes (2). Whilst the definition of active epilepsy (meaning a current diagnosis) includes a history of more than one convulsion, a recent convulsion within last five years and the need to use anti-epileptic drugs (3). The outward effects can vary from wild thrashing movements (tonic-clonic seizure) to milder forms with a brief loss of awareness (absence seizure). Symptoms of an epileptic seizure may include loss of consciousness, diminishing physical strength as well as changes in behavior, temper and mood (4). Diagnosis is typically made based simply on the description of the seizure and surrounding events and the most common anti-epilepticdrugs are Carbamazepine, Primidone, Phenytoin, Phenobarbital and Valproate (5). Underlying causes of epilepsy can include a genetic predisposition, stroke, head injury or ingestion of a toxin (6). Optimizing screening schedules for children to identify symptoms of epileptic seizures is likely to improve morbidity through the use of medications and other prevention programs to avoid an epileptic attack (6). Yet despite the known benefits of these prevention programs, this does not readily occur in clinical care due to a lack of consolidated evidence reporting on the prevalence of epilepsy in Iran. 

In Tanzania, the prevalence of epilepsy was reported to be 10.2 cases per thousand people (7), whilst epileptic recurrence was reported at 5.59 per thousand in another meta-analysis among an Indian population (8). Higher incidences of epilepsy have been reported by some studies (mean of 68.7 per thousand people) though this has been primarily attributed to infectious diseases and premature and/or traumatic births. However, in developed countries epilepsy prevalence is reported as low as 43.4 per ten thousands persons (9). According to one study in Iran published in 2004, prevalence of epilepsy had increased from 2 to 34 per ten thousands persons during the period of 1970 to 2000 (9). This study also identified that epilepsy increases among people older than 65 years of age and among people of Asian descent (9). However, the results from this review are now somewhat dated, particularly considering that a number of publications relating to the prevalence of epilepsy in Iran have been produced in recent years. In light of this gap, we aim to perform a systematic review and meta-analysis of the latest evidence to quantify the prevalence of epilepsy in Iran, which will include sub-group analyses based on region, age and risk factors. This meta-analysis will also be used to validate the results of the existing reviews and has the potential to inform the next phase of clinical practice and preventive medicine.

## Materials & Methods

Literature Search: Systematic review of several databases was conducted in June 2013, with no lower limit set for date of publication. Electronic databases including PubMed, Scientific Information Databases (SID), Google, Google scholar, Elsevier and Scopus were searched for articles published in Iranian that examined the subject of epilepsy. The search strategy included a combination of several key words which were entered into the search fields including: 1) epilepsy AND prevalence AND Iran, 2) epilepsy 3) seizure AND prevalence AND Iran.

Selection of studies and data extraction: Initial study screening was performed by two reviewers and all potentially relevant studies were screened independently by two review authors to assess eligibility for inclusion within the review. All observational studies were considered for inclusion. The primary outcome of interest was the prevalence of epilepsy in Iran, with secondary outcomes examining geographical region, age and epileptic risk factors. Studies that only reported epilepsy management or treatment options were excluded. Data extraction of included studies was performed independently by two review authors using a standardized data extraction template. This included author name, title of article, year and location of study, sample size, gender, prevalence of epilepsy (separated by men and women when reported), age and epilepsy risk factors.

Statistical Analysis: Meta-analysis was performed using method of moments with a random effects model [reported as Effect Estimates (ES) with a 95% confidence interval (CI)] to take into account variances expected between the differing study characteristics. Variance for each study was calculated using the binomial distribution formula. The presence of heterogeneity was determined by the DerSimonian and Laird method and the Breslow-Day test. The I2 statistic was used to examine the difference for between study variability due to heterogeneity rather than chance, with a range from 0 to 100 percent (values of 25%, 50% and 75% are considered to represent low, medium and high heterogeneity respectively). A value of 0% indicates no observed heterogeneity while 100% indicates significant heterogeneity. Significance was set with = 0.1 for the I^2^ statistic to estimate inconsistency between studies. The presence of heterogeneity was further through subgroups analysis and meta-regression. Statistical analyses were conducted using the Statistical Software Package (STATA) version 11.1 for the subgroups of risk factors including psychological, somatic and convulsive diseases in age ranges of 0-20 and greater than 20 years.

## Results

**Fig 1:**
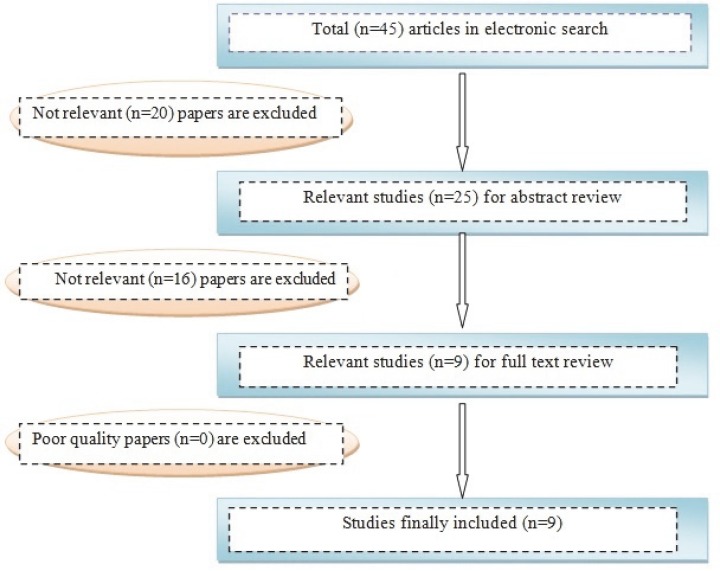
Results of the systematic literature search

A total of 45 studies were identified from the literature search, 25 of these underwent full text review and nine were assessed as meeting all the criteria for inclusion within the review (Figure 1). The nine eligible publications reported on the prevalence of epilepsy in Iran during 2002 and 2010 with a total combined sample size of 7,723 (mean 858 subjects per article). All included studies employed cross-sectional designs. The characteristics of each study are presented in Table 1. 

**Fig 2:**
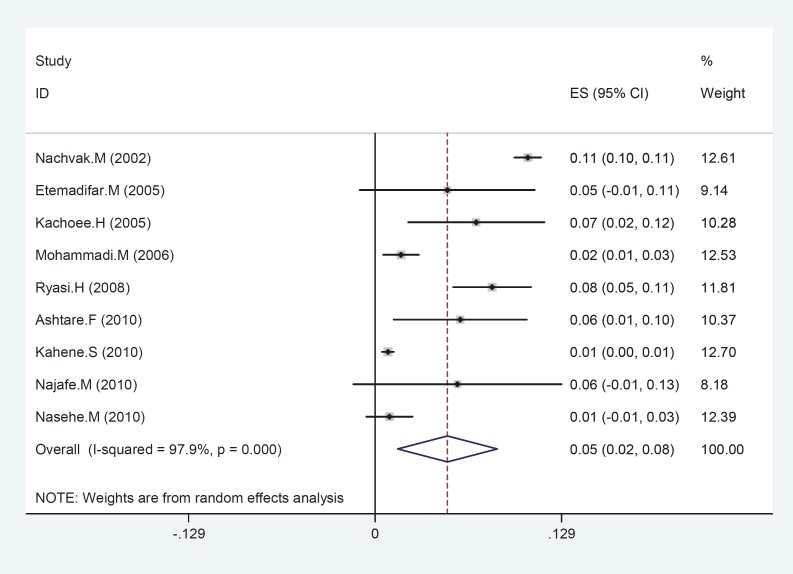
Partial and total epilepsy prevalence using the random effects model. Each square represents the effect estimates for each individual study. Their confidence interval for epilepsy prevalence is reflected by the size of each square proportional to the weight assigned to each study within the meta-analysis. The diamond represents the overall pooled results

**Fig 3:**
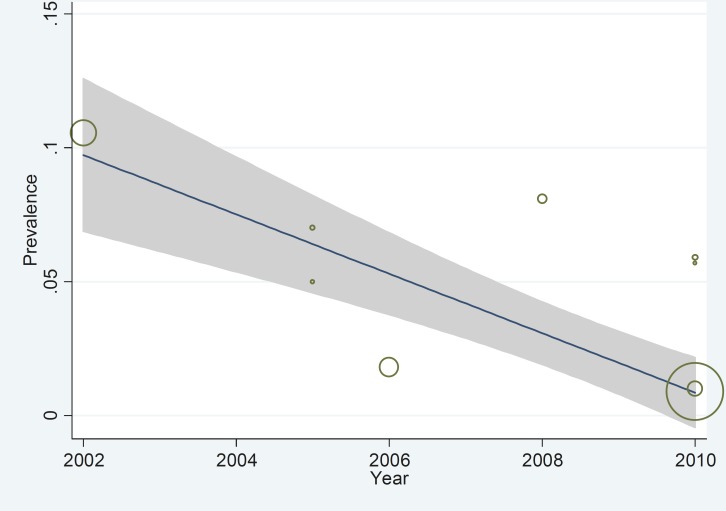
Decreasing trend of epilepsy prevalence in the period between 2002 and 2010 in Iran

Due to the presence of significant heterogeneity (I^2^= 97.9%), meta-analyses were conducted using the random effects model as depicted in Figure 2. The overall prevalence of epilepsy observed across the nine studies was 0.05 (95%CI 0.02 to 0.08; I^2^=97.9%; p= 0.004). Among individual studies Tehran was found to have the highest rate of epilepsy (105 per one thousand people) compared to Brigand with the lowest (9 per one thousand people). The prevalence of recurrent epilepsy is shown in Figure 3 with a decreasing trend observed from 2002 to 2010. 

**Fig 4:**
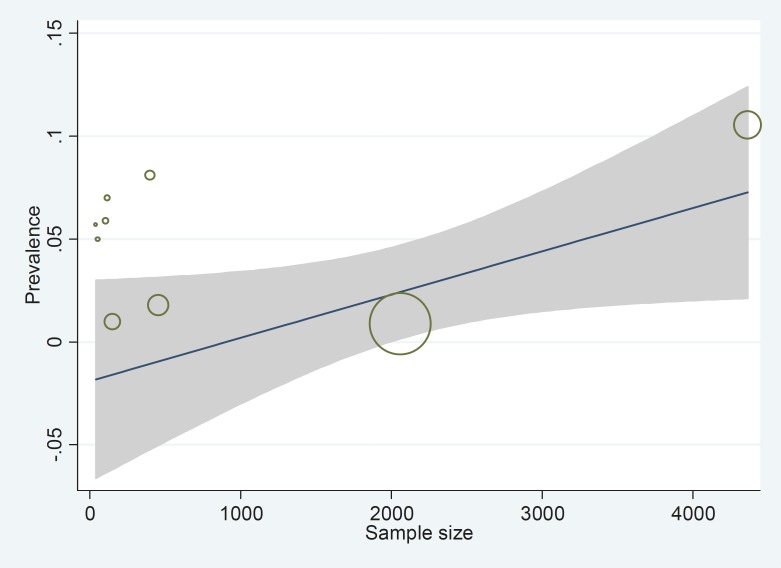
Presentation of the relationship between sample size and epilepsy prevalence. Each circle represents the sample size and as the volume of each circle increases, the sample size is increased

**Fig 5:**
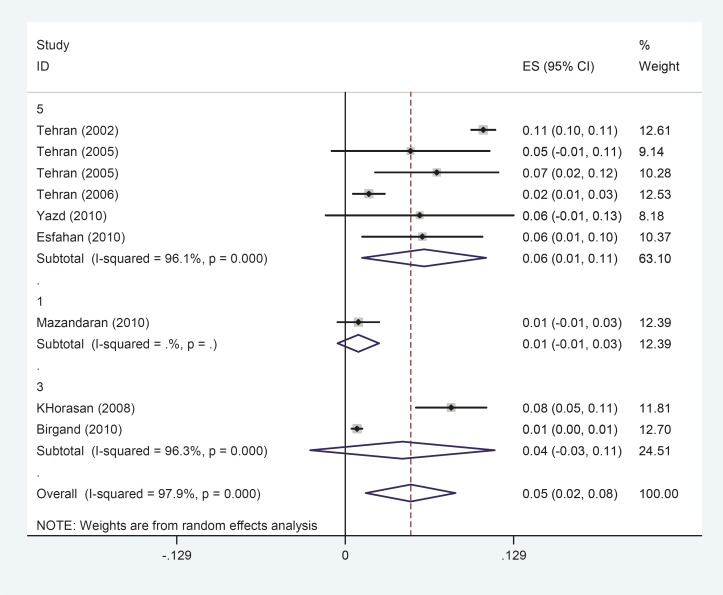
Regional distribution of epilepsy in Iran. Each square represents the effect estimates for each individual study. Their confidence interval for epilepsy prevalence is reflected by the size of each square proportional to the weight assigned to each study within the meta-analysis. The diamond represents the overall pooled results

**Fig 6:**
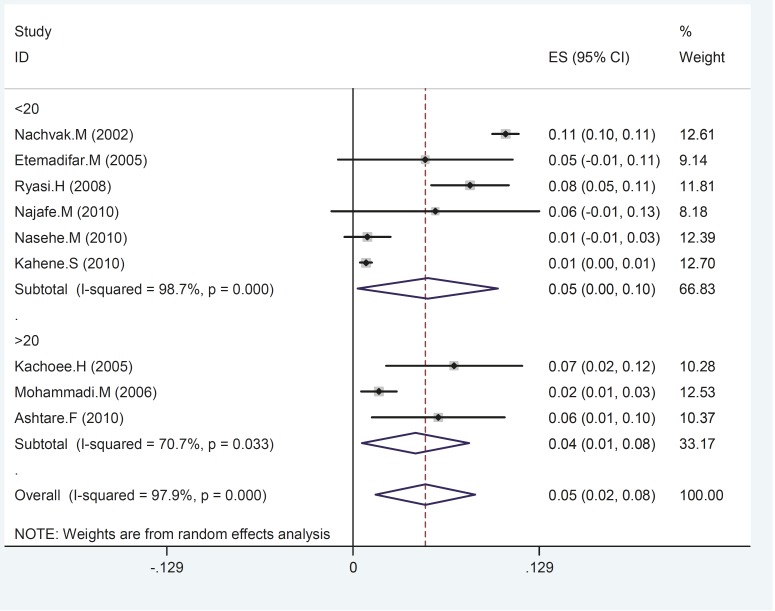
Age distribution of epilepsy in Iran. Each square represents the effect estimates for each individual study. Their confidence interval for epilepsy prevalence is reflected by the size of each square proportional to the weight assigned to each study within the metaanalysis. The diamond represents the overall pooled results)

Table 2 presents percentages of the four risk factors found to cause epilepsy across the nine included studies. The most dangerous risk factor was identified as somatic diseases (39%) whilst hereditary causes were the least common (26%). The prevalence of epilepsy between men and women was found to be similar between genders and across studies. Meta-regression provides one possible reason for the variability observed across the nine studies, with a larger sample size producing higher epilepsy prevalence rates (Figure 4). Moreover, epilepsy distribution across the five definitive regions in Iran (north, south, west, east and central) may also be responsible for the variability observed between results (ES =0.05; 95%CI 0.02 to 0.08; p= .000; Figure 5). Epilepsy recurrence based on age groups are reported in Figure 6, with prevalence estimates of 0.05 (95%CI 0.00 to 0.1; p=0.000) and 0.04 (95CI% 0.01 to 0.08; p= .000) for people less than or equal to 20 years (reported across six studies) and over 20 years of age (reported across three studies) respectively (ES 0.05; 95%CI 0.02 to 0.08; p= 0.033).

## Discussion

The present study has found that the overall prevalence of epilepsy in Iran is 5%, with estimates of 3% for individuals under 20 years of age and 6% for those over 20 years. However, some papers (10, 11) have reported higher rates of epilepsy for people under 20 years with one possible explanation being evaluation of populations using smaller sample sizes. Results from other studies (12, 13, 14, 15) have produced similar findings to our results for people aged over 20 years. A similar metaanalysis in India found epilepsy prevalence to be 5.59 per 1000 persons (8). However, more data was available to assess the outcome for the Indian study with a total pooled sample size of 50,000, in comparison to 7,723 subjects in our study. Based on the available data we can thus assume that the rate of epilepsy in Iran appears is higher than estimates in India.

The Nagchvak et al. (2002) study was found to have the highest rate of epilepsy (0.1056) in a sample size of 4,361 (9) and the Kaheni et al. (2010) study had the lowest (0.009) in a sample of 2,058 people (15). Of the four risk factors known to cause epilepsy, somatic diseases were found to be the most likely to cause an epileptic episode followed by seizures, psychological disorders and genetic predisposition. These risk factors have been reported across several studies including Etemadi Far et al. (2005), Pashapour et al. (2001), and Rezaeei et al. (2000) (10, 12, 16). When considering epilepsy prevalence by geographical regions, higher estimates were obtained for central, eastern and northern Iran, which is similar to findings from other publications (6). This review has several limitations including the presence of substantial heterogeneity (97.9% according to the I^2^ statistic). In an attempt to explore the causes of this variance, we considered the rates of epilepsy whilst adjusting for risk factors (seizures, genetic predisposition, somatic and psychological diseases), age (0-20 and greater than 20 years) and geographical region in Iran (north, west, east, south and central). In addition, a random effect model was employed within all meta-analyses to adjust for the expected differences between studies (caused by examination of a diverse population across a large geographical region). Another limitation relates to a lack of methodological rigor due to inclusion of observational studies and as such selection bias is inevitable. Moreover, our analysis is limited to only those variables reported within the included studies, further restricting the strength of this evidence. Reporting on epilepsy status (being stable or unstable epilepsy) was not reported among studies with results reported as an overview of the population, restricting the generalizability of findings. None of the included studies reported data on medication efficacy and prevalence was not reported across all regions of Iran for every study. Finally, the data is limited only to published studies and those written in Iranian. It is possible that government evaluations and other non-published data may have been inadvertently excluded. 

**In conclusion**, although epilepsy in Iran was found to be much higher than that in other countries, we did observe a decline in prevalence over time. Considering this finding, improved reporting of current estimates are needed to provide more accurate data on epilepsy prevalence, which is necessary to determine if the trend observed in this study is being maintained.

**Table 1:** Features of Different Regions Studies of Iran

**City**	**Study Period**	**Sample size**	**Prevalence per 1000 persons**
Tehran	2005	50	50
Tehran	2002	4361	105.6
Yazd	2010	40	57
Tehran	2005	113	70
Mazandaran	2010	150	10
Esfahan	2010	101	59
Birjand	2010	2085	9
Tehran	2006	454	18
Khorasan	2008	369	81
Total		7723	50

**Table 2:** Prevalence of Risk Factors of Epilepsy in Iran

**Risk factors**	**Subgroups**	**No. of studies**	**Sample size**	**Prevalence % (95%CI)**	**Heterogeneity** ** I** ^2^ **P**	
Risk Factors	Seizure ^1^*	3	264	(65-11) 38	96.1	0.006
	Mental disease 2*	3	617	(57-15) 36	96	0.001
	Somatic disease 3*	4	745	(62-15) 39	98.3	0.001
	Heredity 4*	4	341	(42-9) 26	92.8	0.002

1* =Including fever, tonic-colonic convulsions and epilepsy

2* =Depression, Syncope, Hysteria, Dementia, Stress, bipolar disorders, sleep

3* = Brain diseases, trauma, surgery, tumor, systemic disease, premature infant

4* = Family history

**Table 3:** Regional Epilepsy Prevalence and Age Groups in Iran

**Risk factors**	**Subgroups**	**No. of studies**	**Sample size**	**Prevalence** **(%95) CI** **)**	**Heterogeneity** **I** ^2^ **% P**	
Region	Center	6	5119	(11-1)6	96.1	0.013
	East	2	2454	(8-2)5	97.9	0.224
Age group	North	1	150	(0-3)1	-	-
	0-20 year	3	2604	(8-2)5	92.6	0.053
	20<year	5	5006	3(0-6)	96.9	0.033
